# Supplementary material for: The hidden structure of human enamel
Source: Nat Commun. 2019 Sep 26;10:4383. doi: 10.1038/s41467-019-12185-7 (PMC6763454; doi:10.1038/s41467-019-12185-7)
Supplement: Supplementary file 1 — Supplementary Information [file 41467_2019_12185_MOESM1_ESM.pdf]

Supplementary Information

for

The hidden structure of human enamel

Elia Beniash[*1-4], Cayla A. Stifler[*5], Chang-Yu Sun[5], Gang Seob Jung[6,7], Zhao Qin[6,7], Markus J. Buehler[6,7#], Pupa U. P. A. Gilbert[5,8†#]

[1]Department of Oral Biology, School of Dental Medicine, UPitt, Pittsburgh, PA 15261, USA

[2]Department of Bioengineering, Swanson School of Engineering, University of Pittsburgh, Pittsburgh, PA 15261, USA

[3]Center for Craniofacial Regeneration, School of Dental Medicine, UPitt, Pittsburgh, PA 15261, USA

[4]McGowan Institute for Regenerative Medicine, School of Dental Medicine, UPitt, Pittsburgh, PA 15261, USA

[5]Department of Physics, UW-Madison, WI 53706, USA

[6]Department of Civil and Environmental Engineering, Massachusetts Institute of Technology Cambridge, MA 02139-4307, USA

[7]Laboratory for Atomistic & Molecular Mechanics, Massachusetts Institute of Technology Cambridge, MA 02139-4307, USA

[8]Departments of Chemistry, Geoscience, Materials Science and Engineering, UW-Madison, WI 53706, USA

[*] These two authors contributed equally

[#] Correspondence to: pupa@physics.wisc.edu and mbuehler@mit.edu

[†] previously publishing as Gelsomina De Stasio.

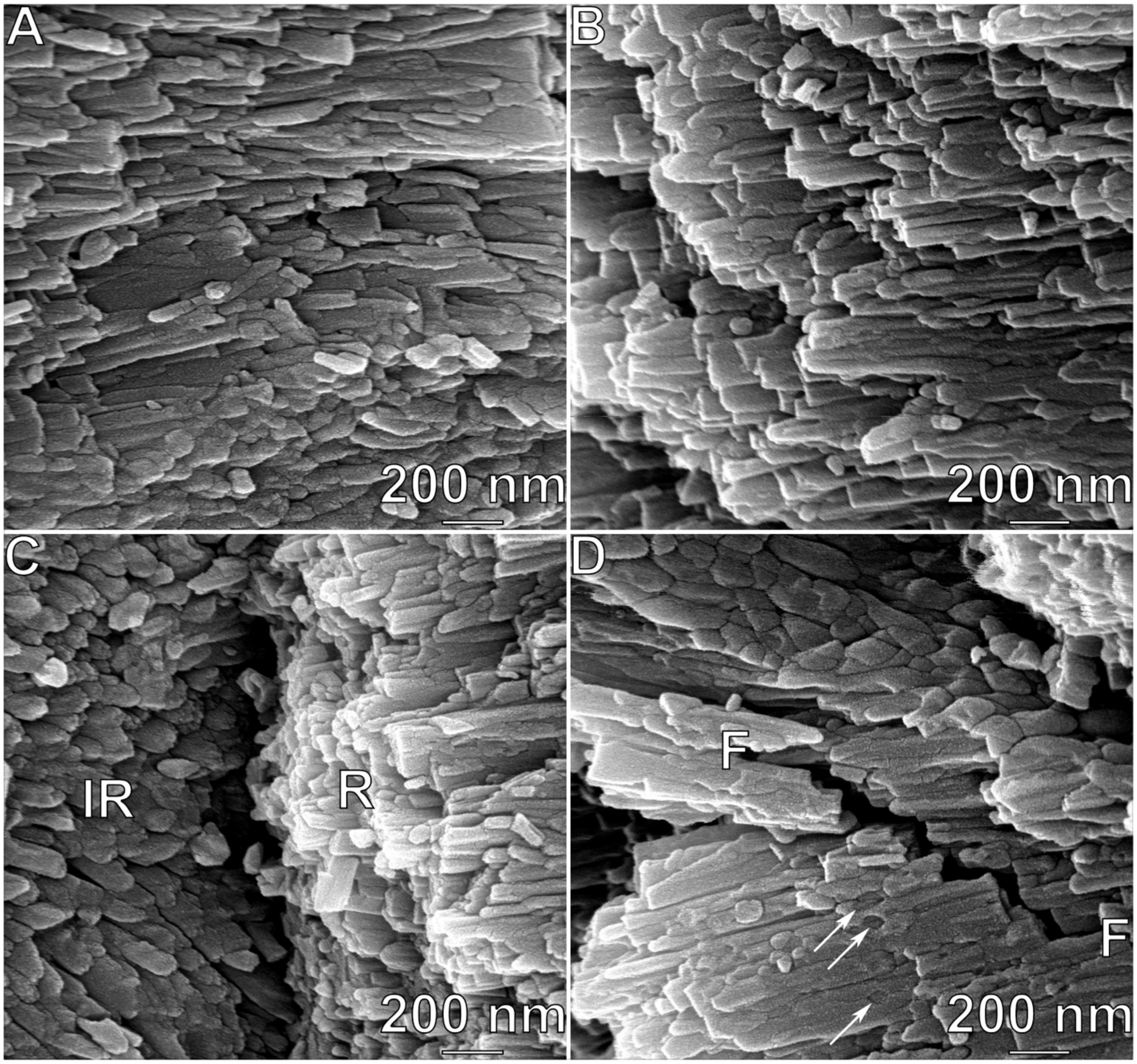

Supplementary Figure 1. SEM images of nanocrystals in human enamel. The enamel sample was cryo-fractured in liquid nitrogen to highlight the morphology of the crystals, independent of water or organics contributions. A. Fractured interrod. B. Fractured rod. C. Fractured rod (R) and interrod (IR) crystal terminations exposed by the removal of the rod. Rod and interrod crystals are irregularly shaped in cross-sections, in agreement with prior observations in mature rat enamel [1]. Notice the rounded tips of most IR crystals, which were not fractured, they terminated with rounded tips at the interface with the next rod. D. Fractured rod showing a zig-zag fracture (from F to F) and rounded holes (arrows) in outer rod crystals, corresponding in shape and size to the nearby rounded interrod crystal tips in C. A few interrod crystals, therefore, may have formed first, and during the maturation process the outer rod crystals grew around and embedded them. (Alternatively, these round holes may be in the organic sheath surrounding the rod, but in this case, they should appear much darker in SEM-secondary electron imaging, thus we think they are crystalline.)

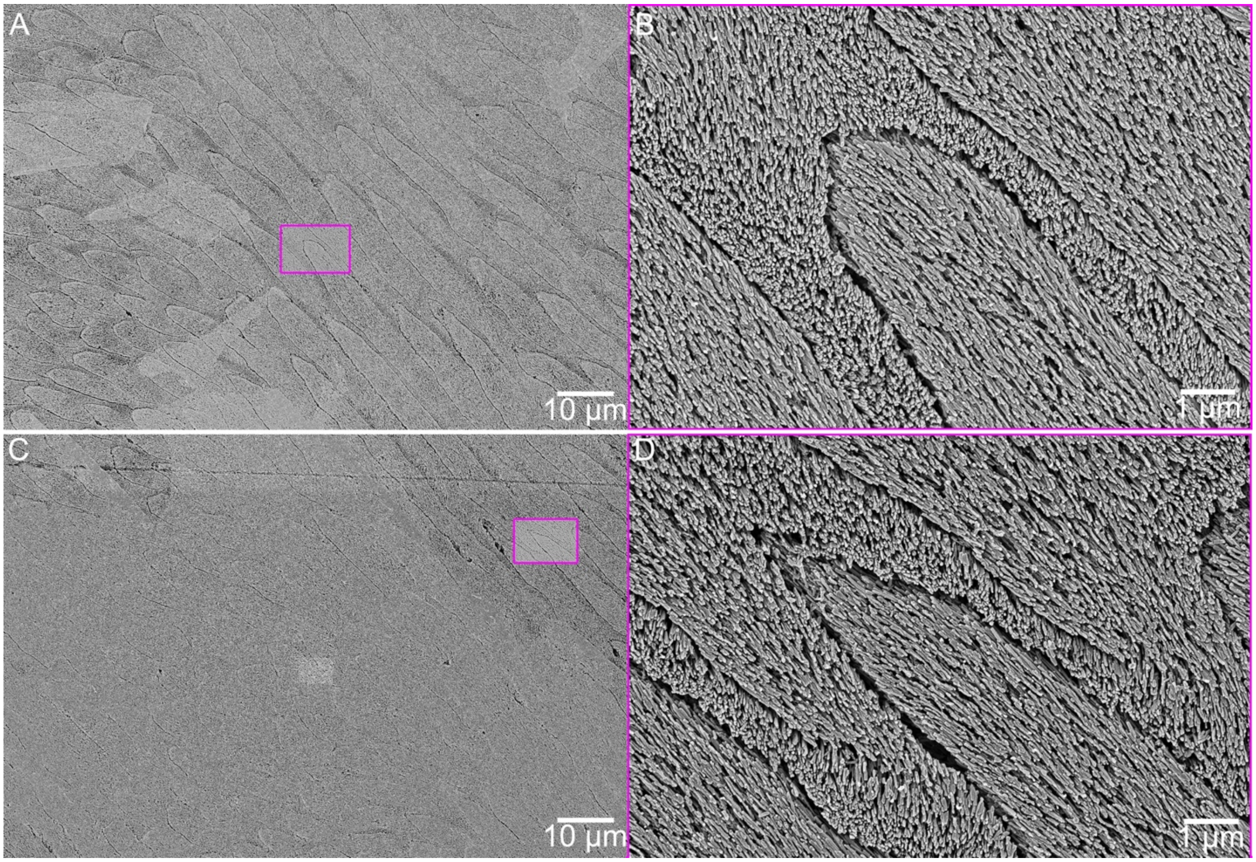

Supplementary Figure 2. Human enamel polished and etched for 2 hours in EDTA. A, C. Low magnification (1,000x) interspersed rod and interrod enamel. The magenta boxes indicate the positions of the images in B and D, respectively. B, D. Higher magnification (10,000x) images of one rod with rounded edge (B), and one with pointed edge (D) surrounded by interrod. The rods in B and D are representative of all rods, observed after etching in this and three additional samples. All crystals within each rod (head) elongate parallel to one another. In Figure 2 all rods show at least a 30° spread in crystal orientations in locations equivalent to those shown here. Such spread is absent from these and all other rods observed by SEM after etching. All crystals are less than 50 nm in width due to thinning during etching.

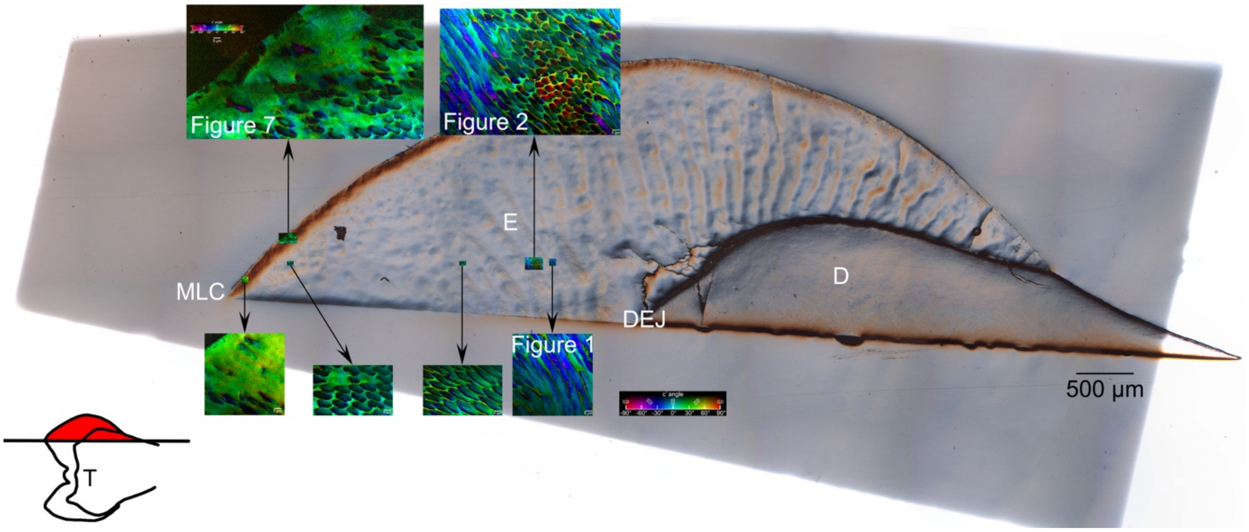

Supplementary Figure 3. Visible light microscopy image of the Hs1 young adult molar tooth analyzed in PEEM. The drawing at bottom left is an outline of the entire tooth (T) cut to expose the buccolingual surface in the plane of the image, further cut along the horizontal line, then the part filled in red was embedded, polished, and coated for PEEM analysis. After polishing enamel (E), dentin (D), the dentin-enamel junction (DEJ), and the mesiolingual cusp (MLC) are all easily distinguishable. The accurate positions and sizes of all 60µm-wide areas analyzed are indicated in the tooth at the same scale as the tooth. Arrows point to magnified PIC maps. Partly overlapping PIC maps formed larger maps, e.g. the map of aprismatic enamel in Figure 7, and inner enamel in Figure 2, which are 4 $\times$ 2 areas and 3 $\times$ 2 areas, respectively. The white region surrounding the entire tooth section is coated with 40 nm Pt, the gray region including the tooth is coated with 1 nm Pt.

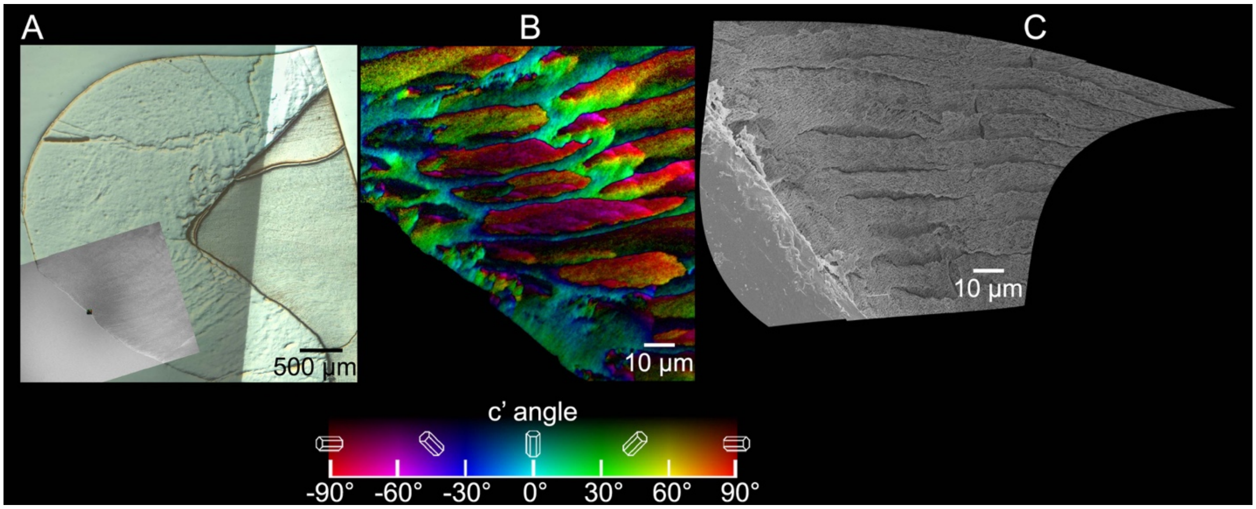

Supplementary Figure 4. Comparison of the same area of the Hs2-3 young adult molar tooth imaged with three methods. (A) visible light microscopy (VLM), SEM (gray level image), and PIC mapping (small colored image at the center of the SEM image), (B) PIC mapping in PEEM, and (C) SEM. A more extensive image of the same tooth and how it was cut is shown in Figure S5. The VLM image and the PIC map were acquired before the tooth was etched, the SEM images in A and C after it was etched. In order to make the images overlap it was necessary to warp the SEM image. This warping was done so the rod magnified in Figure 3 corresponded more accurately than the surrounding ones.

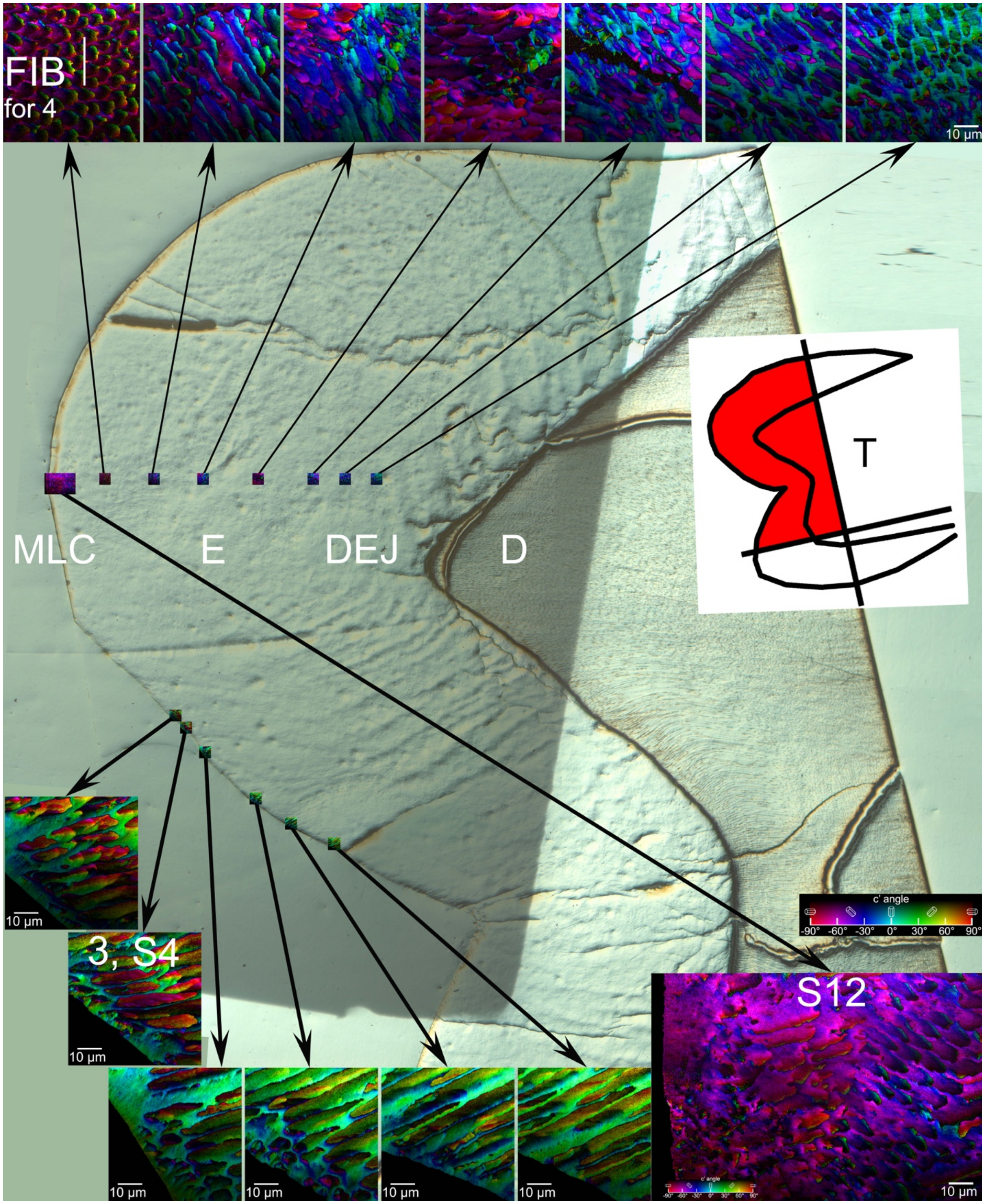

Supplementary Figure 5. Another young adult molar, Hs2-3, also analyzed in PEEM. The drawing on the right is an outline of the entire tooth (T) cut to expose the mesiodistal surface in the plane of the image, further cut along the straight lines, then the part filled in red was embedded, polished, and coated for PEEM analysis. After polishing enamel (E), dentin (D), the dentin-enamel junction (DEJ), and the mesiolingual cusp (MLC) are all easily distinguishable.

The accurate positions and sizes of all 60µm-wide areas analyzed are indicated in the tooth at the same scale as the tooth. Arrows point to magnified PIC maps. The white line on top left (FIB) indicates the area cut by FIB for the TEM experiments in Figure 4. The maps of aprismatic enamel in Supplementary Figure 12A,B,C are 3 × 2, 2 × 2, and 3 × 2 maps, those in Supplementary Figure 12D-I are single 60µm-wide maps from this tooth. PIC maps from a transect of the entire enamel layer, sampled every 300 µm, are shown at the top. Notice the magenta (-60°) orientation of the aprismatic enamel, which is also shared by the outer- and middle-enamel interrod. The inner enamel interrod, instead, gradually rotates from cyan (0°) to green (+30°) as shown in the last 3 PIC maps of the transect (top right).

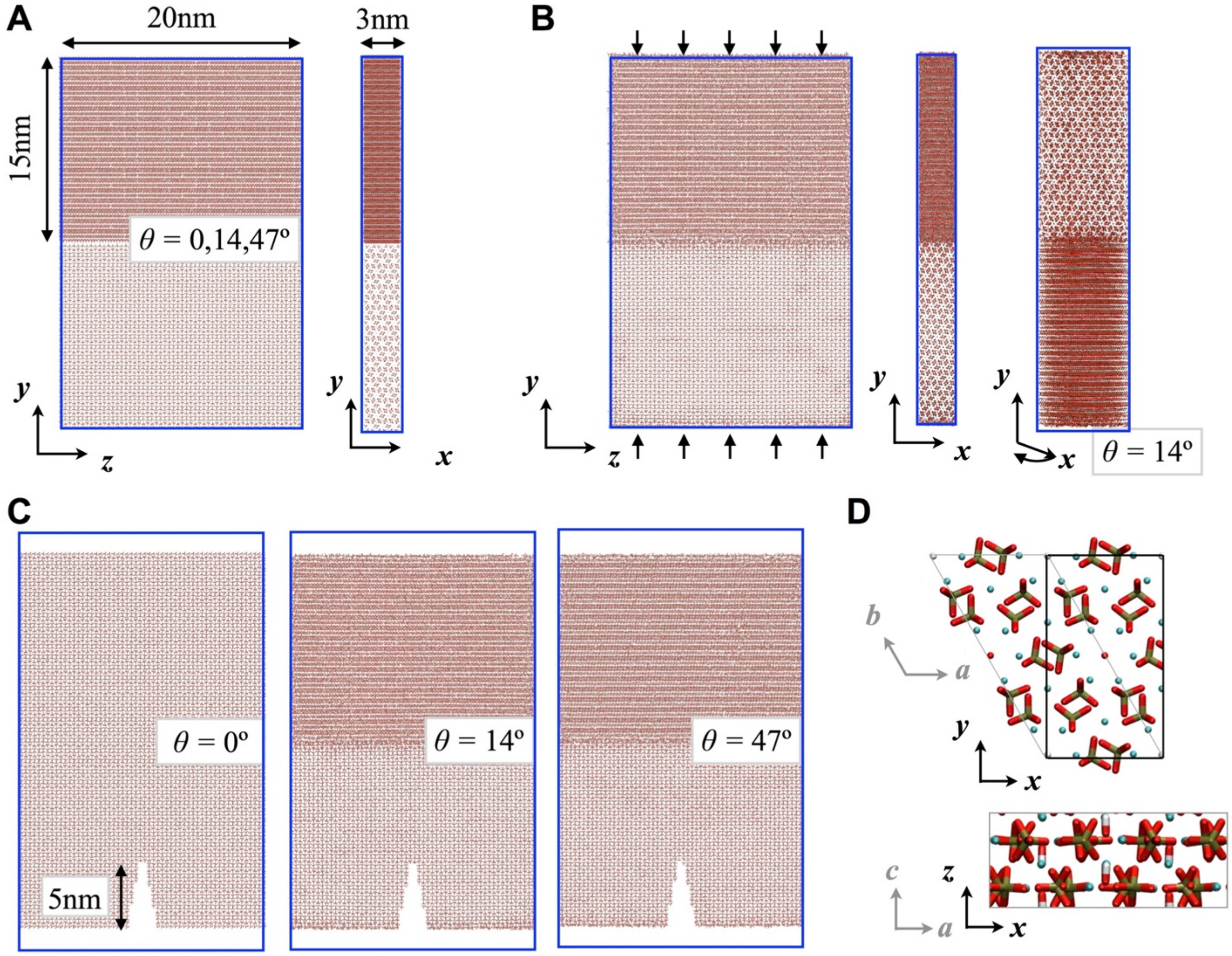

Supplementary Figure 6. Molecular dynamics (<D) simulations of bicrystals, with their *c*-axes mis-oriented by 0°, 14.1°, and 47°. (**A**) The initial structure of two crystals (20 nm × 15 nm × 3 nm) with periodic boundary conditions (Blue boxes indicate the simulation boxes). Vacuum was inserted between two crystals before sintering. (**B**) Snapshots of 14° *c*-axis mis-oriented bi-crystals after the sintering process (arrows indicate the direction of the high pressure for the sintering process). Mis-orientations θ are measured as angles of rotation around the *y* axis, with θ = 0° when the *c*-axis is along z. The crystal structures of both top and bottom HAP crystals are well preserved after sintering and structural relaxation. (**C**) An initial crack (~5 nm) was inserted after the simulation box stretched to have vacuum space (20 Å) between image cells in the *y* direction. (**D**) Schematic for the relation between the materials direction and coordinates.

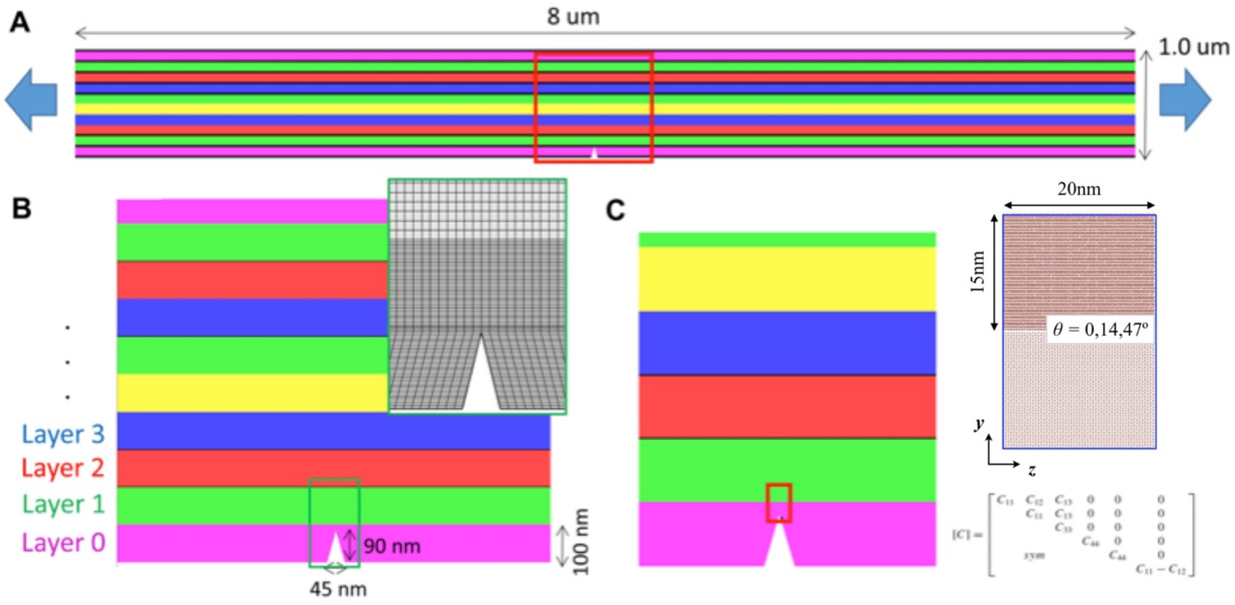

Supplementary Figure 7. Finite Element Method (FEM) model of a multilayered HAP structure. (**A**) The 10 layered HAP structure with overall 8 µm × 1 µm dimension for FEM simulation. (**B**) Each HAP layer is 100 nm and the triangular crack is at the bottom, in the middle of the first HAP layer (Layer 0). (**C**) The constitutive law of each layer of the FEM is defined according to the measurement based on HAP molecular MD model and the deformation field at the crack tip of the FEM was measured to thereafter provide the input as a more realistic boundary condition for MD simulations of fracture test.

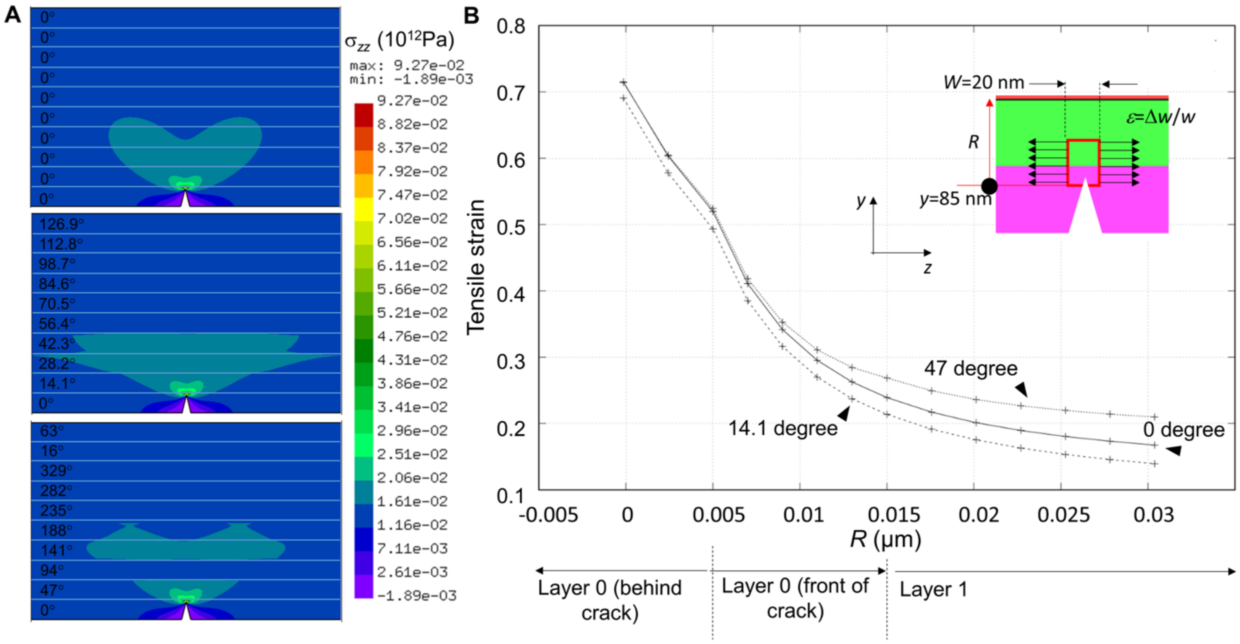

Supplementary Figure 8. Result of FEM simulations at various mis-orientations. (**A**) Distribution of the tensile stress along the loading direction around the crack tip for mis-orientation of 0º, 14.1º and 47º (from top to bottom) with each element colored according to the stress state defined by the color legend on the right. The grain boundaries are indicated by lighter blue parallel lines and the material orientation angle is indicated for each layer in the figure. Notice that layer 2 of the 47º case has lower stress than the neighboring layers (11.6~16.1 GPa versus 16.1 to 20.6 GPa) simply because of the lower modulus along the loading direction for the material orientation at 94°, and the continuity requirement of the displacement field (no slip at the grain boundaries). (**B**) Summary of the tensile strain within a 20 nm × 30 nm region around the crack tip for the three mis-orientation angles near the interface of two HAP layers.

Notice in A that for the case of small angle mis-orientation (14°) the total area of the significantly deformed material (with σ_{zz}=16.1 to 20.6 GPa) is larger than that of the homogeneous HAP (0°) and at the same time such configuration reduces the concentrated deformation at the crack tip , as shown in B, demonstrating that 14° mis-orientation leads to more uniformly distributed stress at the crack tip under loading. This phenomenon is mainly caused by the fact that the distribution of stress at the crack tip depends on the heterogeneous modulus along the pulling direction because all the elemental deformation is subject to no-slip condition at the grain boundaries, before material failure at the extreme condition. Moreover, such heterogeneity for different layers is caused by the anisotropic feature of HAP, making some layers much softer than others along the loading direction. Again, at 47° mis-orientation, A shows that layer 2 has a much lower stress because this layer extends in the ~90° direction, which is much softer than the 0° direction, thus the amount of significantly deformed material is reduced, yielding a higher deformation at the crack tip, as shown in B.

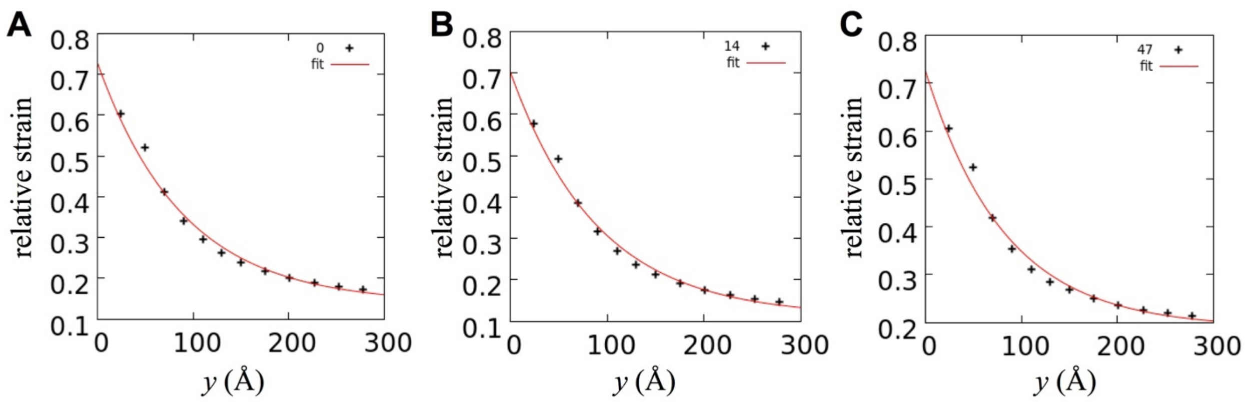

Supplementary Figure 9. Relative strain vs. position curves at various mis-orientations. (**A-C**) The inhomogeneous deformation from FEM results and fitted functions for model with mis-orientation angles of 0°, 14.1°, and 47°.

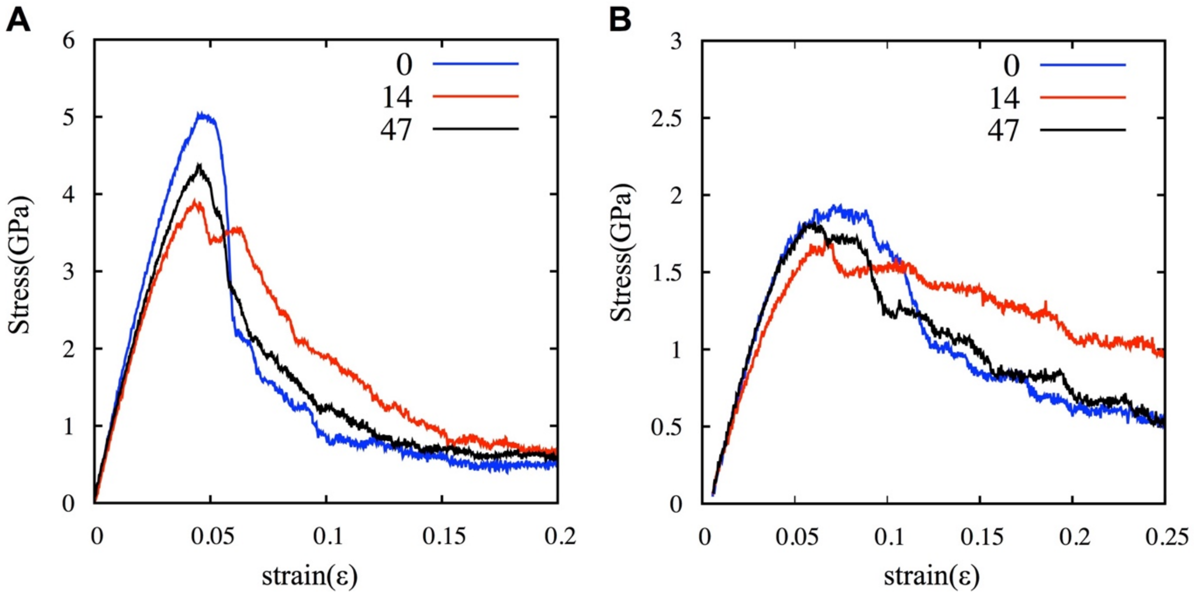

Supplementary Figure 10. Stress-strain curves at various mis-orientations. (**A**) The stress-strain curves of three bi-crystal systems (θ=0°, 14.1°, and 47°) under homogenous tensile loading along *z* (see Supplementary Figure 6 for the orientation of axes). (**B**) The stress-strain for the three models under the inhomogeneous loading, again along z based on FEM.

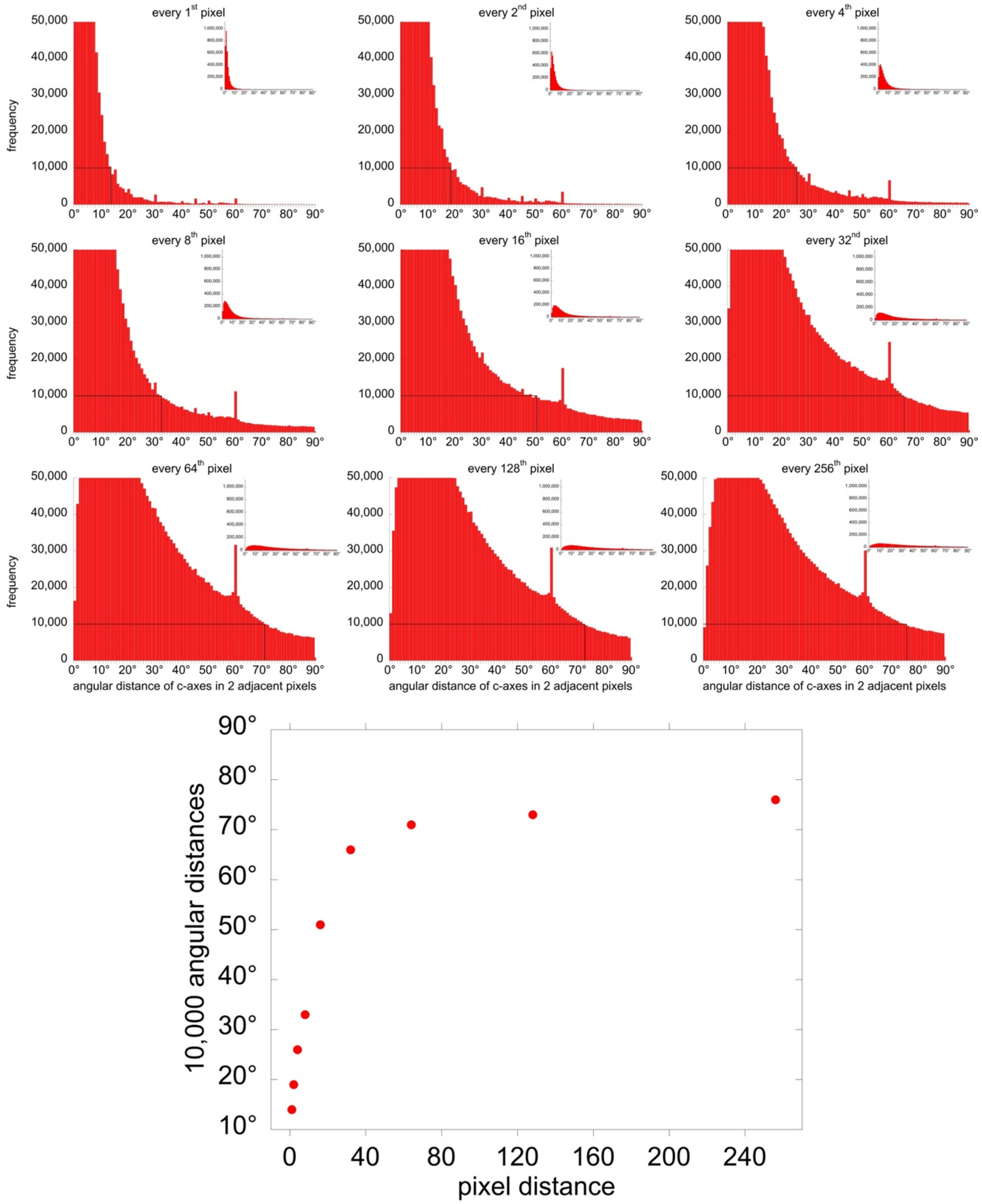

Supplementary Figure 11. Angular distances between crystalline *c*-axes in two pixels, adjacent or at various distances. Histograms of angular distances between *c*-axes in 2 pixels, measured across all of Figure 2, and sampled at every 60-nm pixel (as in Figure 6), every 2nd, 4th, 8th, 16th, 32nd, 64th, 128th, and 256th pixel. Comparison of these histograms demonstrates that changes in crystal orientation are gradual. In the first histogram, most pixels (and crystals in enamel) are oriented 0°-30° apart from their neighboring crystals, with maximum at 1°. The spike at 60° corresponds to rod-interrod edges where no organic sheath is present, e.g. at blue-green head-

tail interfaces. (Organic sheaths at many of the rod-interrod interfaces appear black in PIC maps, as they are not polarization dependent). The overwhelming majority of pixels, however, change orientation gradually, and are within 30° of their neighbors. The plot shows the angular distances measured in the above 9 histograms at 10,000 counts, which increases monotonically because all changes are gradual, therefore the greater the spatial distance between sampled pixels the higher the likelihood that their angular distance is greater.

The insets show the same histograms zoomed-out. They are all displayed on the same vertical axis, ranging from 0 to 1,000,000, and show the peak frequency decrease, as expected for decreasing number of sampled pixels.

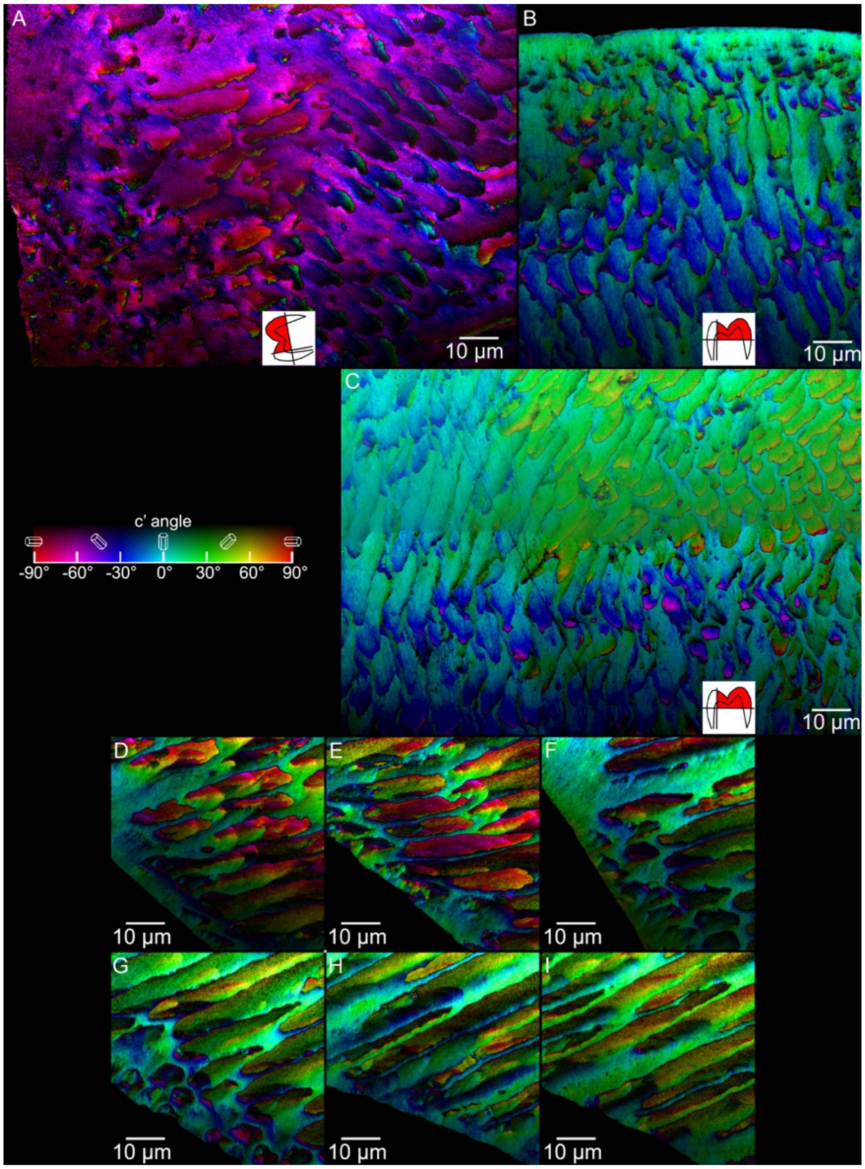

Supplementary Figure 12. PIC maps of the same tooth as in Supplementary Figure 5. The tooth sample H2-3 is mounted and analyzed horizontally (A), and then vertically (B). Notice the consistent color of the aprismatic enamel, which is magenta (-60°) in the A and green (+30°) in the B PIC maps, thus for a 90° rotation of the sample the *c*-axis angles detected by PIC mapping are precisely orthogonal. The area in C was acquired in mid-enamel, 600 µm from the cusp, and confirms a 30° or greater angle spread within each rod. D-I. PIC maps of aprismatic enamel acquired below the cusp of the tooth in Supplementary Figure 5. The areas in A, B, C are 3 × 2, 2 × 2, and 3 × 2 maps, respectively, those in D-I are single 60µm-wide areas. Another surprising observation in Figure 7 and here is that the crystal *c*-axis orientation in aprismatic enamel is not perpendicular to the tooth surface but approximately parallel to it in Figure 7.

Supplementary Table 1. Summary of the lattice parameters of HAP. The data from experiments and Density Functional Theory (DFT) calculations are obtained from Corno *et al.* [2]. Schematics of lattice parameters (*a* and *b*) are shown in Supplementary Figure 6D.

Lattice	Experimental (Å)	DFT (Å)	Current MD (Å)
a	9.41-9.43	9.11-9.56	9.35
c	6.88	6.83-6.95	6.9

Supplementary Table 2. Summary of the elastic constants of HAP. The data from experiments and DFT calculations are obtained from Menéndez-Proupin *et al.* [3].

Elastic constant	Experimental (GPa)	Theory (GPa)	Current MD (GPa)
C11	123-137	117-140	134.5
C33	123-172	175-231	171.3
C44	39.6-50.9	47.5-56.4	50.4
C12	42.5-53	26.2-48.9	48.1
C13	55-59.5	58.3-68.5	65.4
Bulk modulus	82-89	84.6-90	87.4

Supplementary Table 3. The inhomogeneous deformation speed along the z direction as a function of position of *y* from FEM simulations.

	Loading functions (Å/ps)
0°	± (0.587984*exp(-0.0111201*y)+0.139238)*0.05
14.1°	± (0.590435*exp(-0.0110679*y)+0.111772)*0.05
47°	± (0.536366*exp(-0.0121161*y)+0.188909)*0.05

Supplementary References

1 Selvig, K. A. The crystal structure of hydroxyapatite in dental enamel as seen with the electron microscope. *J Ultrastruct Res* **41**, 369-375 (1972).

2 Corno, M., Busco, C., Civalleri, B. & Ugliengo, P. Periodic ab initio study of structural and vibrational features of hexagonal hydroxyapatite Ca10(PO4)6(OH)2. *Phys. Chem. Chem. Phys.* **8**, 2464-2469, doi:papers3://publication/doi/10.1039/b602419j (2006).

3 Menéndez-Proupin, E. *et al.* Computer simulation of elastic constants of hydroxyapatite and fluorapatite. *J Mech Behav Biomed Mater* **4**, 1011-1020, doi:papers3://publication/doi/10.1016/j.jmbbm.2011.03.001 (2011).
